# Competitive Performance, Training Load and Physiological Responses During Tapering in Young Swimmers

**DOI:** 10.2478/hukin-2013-0052

**Published:** 2013-10-08

**Authors:** Argyris G. Toubekis, Evgenia Drosou, Vassilios Gourgoulis, Savvas Thomaidis, Helen Douda, Savvas P. Tokmakidis

**Affiliations:** 1 Kapodistrian University of Athens, Athens, Greece, Faculty of Physical Education and Sports Science, Department of Aquatic Sports, Athens, Greece.; 2 Democritus University of Thrace, Komotini, Greece, Department of Physical Education and Sports Science, Komotini, Greece.

**Keywords:** training loads, tethered swimming force, arm strength, periodization

## Abstract

The study examined the changes of training load and physiological parameters in relation to competitive performance during a period leading to a national championship. The training content of twelve swimmers (age: 14.2±1.3 yrs) was recorded four weeks before the national championship (two weeks of normal training and two weeks of the taper). The training load was calculated: i) by the swimmer’s session-RPE score (RPE-Load), ii) by the training intensity levels adjusted after a 7×200-m progressively increasing intensity test (LA-Load). Swimmers completed a 400-m submaximal intensity test, a 15 s tethered swimming and hand-grip strength measurements 34–35 (baseline: Test 1), 20–21 (before taper: Test 2) and 6–7 (Test 3) days before the national championship. Performance during the national championship was not significantly changed compared to season best (0.1±1.6%; 95% confidence limits: −0.9, 1.1%; Effect Size: 0.02, p=0.72) and compared to performance before the start of the two-week taper period (0.9±1.7%; 95% confidence limits: 0.3, 2.1%; Effect size: 0.12, p=0.09). No significant changes were observed in all measured physiological and performance related variables between Test 1, Test 2, and Test 3. Changes in RPE-Load (week-4 vs. week-1) were correlated with changes in performance (r=0.63, p=0.03) and the RPE-Load was correlated with the LA-Load (r=0.80, p=0.01). The estimation of the session-RPE training load may be helpful for taper planning of young swimmers. Increasing the difference between the normal and last week of taper training load may facilitate performance improvements.

## Introduction

It is suggested that swimmers should follow a taper to increase performance, which is a period of progressively reduced training load several weeks before an important competition (Bosquet et al., 2007). Positive changes of psychometric, physiological, and performance-related parameters have been observed during the taper period (Hooper et al., 1999; Mujika et al., 1996; Papoti et al., 2007). Performance improvement is probably achieved by an appropriately planned maintenance of intensity and reduction of the training load (Mujika and Padilla, 2007).

The calculation of training load has many difficulties and limitations. To estimate the training load in competitive swimmers, however, simple methods have been applied. One of these requires the establishment of training intensity levels based on a speed-lactate curve and subsequent estimation of the training load (Mujika et al., 1996). One more option is the session-RPE method, which has been suggested to be valid for application in swimming (Wallace et al., 2009). Although other psychometric variables have been evaluated during the taper period (i.e. profile of mood state; Hooper et al., 1998), there are no reports for the use of the session-RPE method for the estimation of the training load during a training period leading to a national championship. Furthermore, there are no reports comparing the session-RPE and the blood lactate-based method that have been used for training load estimation.

Changes in performance variables such as tethered swimming force (Hooper et al., 1998; Papoti et al., 2007) or arm-crank power have been related with swimming performance (Trinity et al., 2006). However, tethered swimming forces have been measured before swimming competition simulating tests (Hooper et al., 1998; Papoti et al., 2007) but they have never been tested before official competitions. Additionally, other physiological changes, such as the lactate concentration or muscle power, may occur during the taper period (D’Aquisto et al., 1992; Trinity et al., 2006). These parameters may show a different rate of change in young compared to adult swimmers and there are few reports for the effect of tapering and accompanied physiological and stroke parameter responses on young age swimmers (D’Aquisto et al., 1992). The purpose of the present study was to examine the effect of changes on session-RPE training load in comparison with changes of the training load calculated by the training stimulus on competitive performance. Additionally, changes of tethered swimming force and physiological parameters such as blood lactate, were examined in relation to competitive performance a period before and during taper for the national championship.

## Material and Methods

### Participants

Twelve competitive swimmers (female, n=8, male, n=4, age: 14.2±1.3 yrs, body mass: 62.3±9 kg, body height: 1.72±0.08 m, mean±SD) participated in the study. The stage of biological maturation was assessed at the start and the end of the experimental period (start: 3.2±1.0, end: 3.4±1.0; Tanner and Whitehouse, 1976). Each swimmer had qualified and participated in the Greek national championship (NC) competing in two or three races at various distances within a period of five days (i.e. 50 and 200 or 200 and 1500 m). The same race was used for comparison between competitions. However, because of different race distances the average speed and percentage performance change of all individual races was used in the analysis. Five swimmers were specialized in breaststroke, six swimmers in front crawl and backstroke events, and one swimmer in butterfly. All swimmers had followed regular training and competitive participation during the previous four to six years. The swimmers and their parents were informed in detail about the experimental risks and procedures and signed an informed consent form prior to the investigation. The research protocol was reviewed and approved by the Institutional Review Board for the protection of Human subjects according to the Helsinki declaration.

### Measures

**Figure 1:**
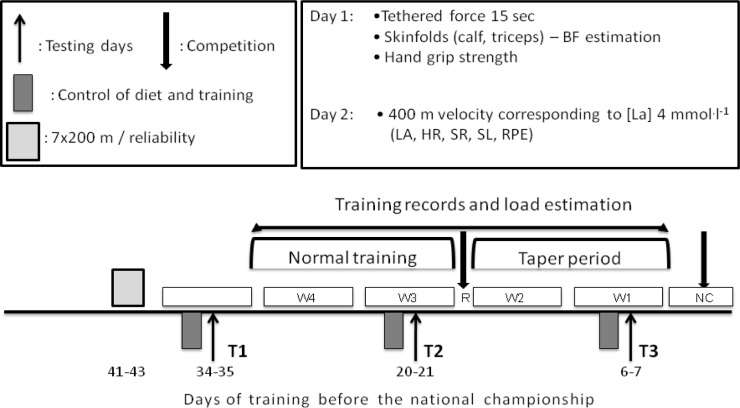
The experimental procedure of the study. BF: body fat, LA: blood lactate, HR: heart rate, SR: stroke rate, SL: stroke length, RPE: rate of perceived exertion, W1, W2, W3, W4: weeks before the national championship, T1, T2, T3: two-day testing periods, R: regional qualifying competition, NC: week of national championship

The study was conducted in a repeated measures design and the time course of testing and training data collection is presented in Figure 1. Forty-one to 43 days before the NC, the swimmers completed 7×200-m repetitions with progressively increasing intensity (Pyne et al., 2001). From the speed-lactate relationship of the second order polynomial function, the velocity corresponding to blood lactate concentration of 4 mmol·L^−1^ (V4) was calculated by interpolation. Subsequently, each swimmer participated in a series of 2-day tests 34–35 days (baseline: Test 1), 20–21 days (before the taper: Test 2), 6–7 days (Test 3) before the NC.

The first testing day consisted of: i) a 15 s tethered swimming test with maximum intensity (MuscleLab, Ergotest) that had been previously tested for reliability (intraclass correlation coefficient: ICC=0.985, p<0.05; Toubekis et al., 2010), ii) measurement of the hand-grip strength of both arms (Grip-D TKK S401 Takei Scientific instruments; ICC=0.967 right and 0.983 left arm, p<0.05), iii) measurement of mid-calf and triceps skinfolds for estimation of the percentage body fat (ICC=0.984, p<0.05) (Slaughter et al., 1988). The force during the 15 s tethered swimming test was averaged every second and the fatigue index was calculated as the percentage change of the peak to end force. The lean body mass was also calculated (Lean body mass = Body mass – Fat mass). The evaluation of Hand-Grip strength was selected because it had been shown to correlate with performance in young swimmers (13 years old, r=0.73; Geladas et al., 2005).

The second testing day included a 400 m submaximal intensity test at a speed corresponding to V4. The time to complete three stroke cycles (C3) was used to calculate the stroke rate (SR=180·C3^−1^). The stroke length (SL) was calculated from swimming speed (V) and stroke rate (SL=V·SR^−1^). The heart rate (HR) was recorded continuously during the 400 m test (Polar S810i) and the rate of whole body perceived exertion (RPE) was recorded before and immediately after the test (Borg, 1970). A finger-tip blood sample (10 μl) was taken at rest and after the 400 m test for the determination of blood lactate concentration (miniphotometer, Dr lange, LP20).

### Procedures

Four weeks preceding the NC the training load was calculated with two methods and was expressed in arbitrary units (AU). For the LA-Load method the training intensity was individually classified in five intensity levels based on the speed-lactate curve and corresponding to a blood lactate concentration (level I: ∼2 mmol·L^−1^, level II: ∼4 mmol·L^−1^, level III: ∼6 mmol·L^−1^, level IV: ∼10 mmol·L^−1^ and level V: maximum intensity). Subsequently, the daily load was calculated by multiplying the distance covered at each level with a corresponding factor of 1, 2, 3, 5, 8 divided by 1000 (Mujika et al., 1996). For the RPE-Load method the swimmers’ training was recorded daily and the training load was calculated multiplying the 10-point rating of the perceived exertion scale (RPE) score that was reported 30 minutes after each training session, with the training duration in minutes (Wallace et al., 2009). After the completion of each training session, the coach rated his perception of the subjective fatigue for each swimmer on the same RPE scale. One of the coaches agreed to provide the session RPE for the total experimental period for the nine swimmers he was responsible for. Therefore, comparison of session RPE of coach and swimmers is for nine swimmers. All swimmers started the taper period fifteen days before the NC (week 2 and week 1). Nine swimmers participated in an official local competition two weeks before the NC competing at the same events as in the NC. The best time of the season for a 50 m swimming pool (the starting list time) and competitive performance in the NC were retrieved by the officially printed times. Two separate comparisons of performance change were made and reported: i) between pre-Taper and NC (n=9) and ii) between season best (the starting list time) and NC (n=12). Two swimmers did not participate in the last testing session (Test 3), one because of injury and another because of personal constrains. Therefore, the 400 m data are for ten swimmers.

### Statistical Analysis

One-way analysis of variance for repeated measures was used to compare the changes on dependent variables. Two-way analysis of variance for repeated measures on both factors was used to compare changes of SR, SL HR, blood lactate, RPE (3 tests × number of repetitions). Multiple comparisons were made using the Tukey HSD post-hoc test. The effect size (ES) was calculated according to Rhea (2004). The Pearson correlation coefficient was used to test the relationship between variables. The data is presented as mean±SD and the level of significance was set at p≤0.05.

## Results

**Figure 2:**
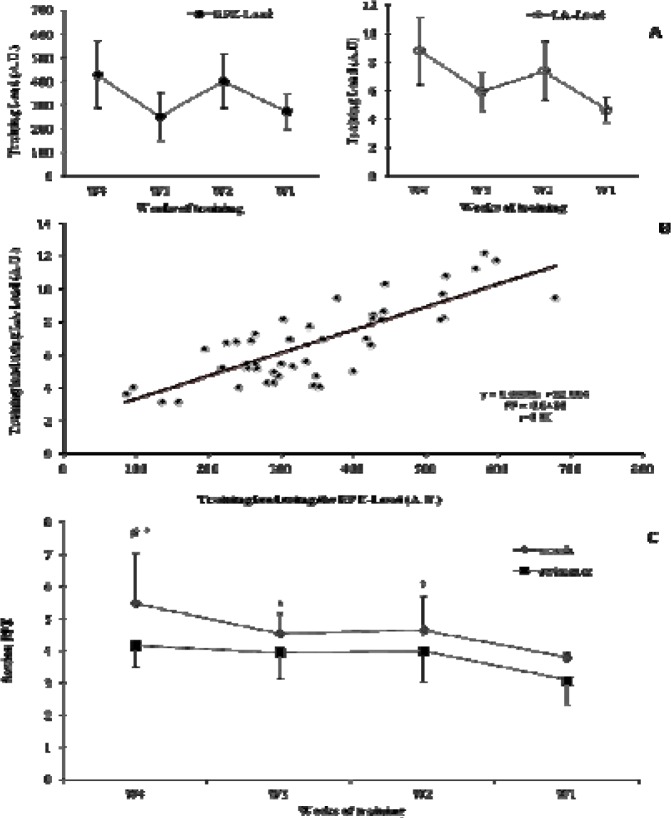
Changes of training load calculated by the session-RPE (RPE-Load) and from the lactate curve (LA-Load) in panel A. The relationship of the two methods used for the estimation of training load in the middle panel B. The changes of mean weekly training rating of perceived exertion (RPE) of the coach and swimmers, *: compared to week 1, #: between coach and swimmers at week 4, (n=9, panel C)

The LA-Load followed the same trend as the RPE-Load during the four weeks and was decreased by 45±11% from week 4 to week 1 (Figure 2A). The lowest RPE-Load was observed during the last week before NC (Figure 2A) and was decreased from week 4 to week 1 by 30±29%, while the training distance was reduced by 35±12% during the same period (W4: 35100±8250 m, W3: 26200±6400 m, W2: 30000±6200 m, W1: 22200±2700 m; W1 vs. W2, W3, W4 and W3 vs. W4 p<0.01). Training loads calculated by the two different methods were significantly related (r=0.80, Figure 2B, p=0.01). The weekly session-RPE perceived by the swimmers was lower compared to the session-RPE of the coach (p=0.02). The post-hoc analysis revealed no difference during week 3, week 2 and week 1, but lower values during week 4 (5.5±1.6 vs. 4.2±0.6, n=9, p<0.05, Figure 2C).

**Figure 3:**
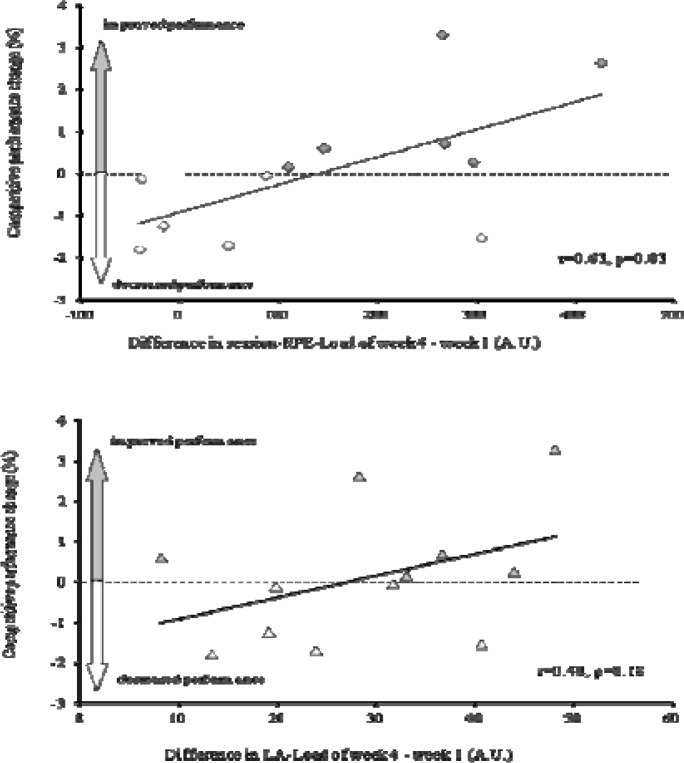
The percentage change of performance with the training load difference of week 4 minus week 1 relationship. The training load calculated from the session-RPE method (RPE-Load, upper panel) and from the speed-lactate curve method (LA-Load, lower panel). The discontinuous horizontal line separates swimmers who were improved (filled dots above the line) with those not improved (open dots below the line)

No significant change during NC compared to the season best performance was observed (starting list time vs. NC: 0.1±1.6%; 95% confidence limits: −0.9, 1.1%). The competitive speed at the starting list compared to the speed at the NC was not different (Start list: 1.33±0.15 vs. NC: 1.34±0.16 m·s^−1^, ES: 0.02, p=0.72). Similarly, the percentage change during the NC compared to performance in the competition before the start of the taper was not significant (0.9±1.6%; 95% confidence limits: −0.3, 2.1%). The average speed of the races at the NC was not different when compared to the speed of the corresponding races performed before the start of the taper (Pre-Taper Competition: 1.34±0.12 vs. NC: 1.35±0.12 m·s^−1^, ES=0.12, p=0.09). The RPE-Load difference of week 4 minus week 1 was correlated significantly with the percentage performance change in the NC versus the starting list (r=0.63, Figure 3, p=0.03). The LA-Load changes were not related significantly to performance changes (r=0.41, Figure 3, p=0.18). No relationship was observed between weekly volume and volume of training performed at the three higher levels of intensity changes (III, IV, V) with performance changes in the NC (p>0.05).

Hand-grip strength, mean tethered swimming force, the fatigue index during 15 s tethered swimming, body fat and lean body mass were not significantly changed during the three testing periods (Table 1). All changes of these variables between tests were not significantly related to the percentage change of performance (p>0.05). The swimmers completed the 400 m test at the same speed in all three testing sessions (Test 1: 1.19±0.11, Test 2: 1.19±0.11, Test 3: 1.20±0.12 m·s^−1^, p>0.05). Blood lactate concentration at the start and immediately after the 400 m test was no different between testing periods (Test 1, start: 1.7±0.3, end: 5.3±1.6; Test 2, start: 1.9±0.4, end: 5.0±1.5; Test 3, start: 1.5±0.3, end: 4.8±1.6 mmol·L^−1^, p>0.05). Percentage change of blood lactate from Test 1 vs. Test 2 was related to performance change at the NC vs. starting list (r=0.63, p=0.05). The HR, SR, SL of the 400 m test were similar during the three testing periods (Table 2). No significant correlation was observed between SR or SL percentage changes and percentage of performance change at NC (p>0.05). RPE was not different between tests (Test 1, start: 0.5±1.0, end: 4.8±1.8; Test 2, start: 0.6±1.0, end: 4.8±1.6; Test 3, start: 0.8±0.9, end: 5.0±1.6, p=0.77).

## Discussion

The young national level competitive swimmers who participated in the present study failed to show significant improvement in performance following a moderate reduction in the training load during a two-week taper. Nevertheless, six out of twelve swimmers showed performance improvement. The two methods used for the estimation of the training load in the present study are significantly related; however, the RPE-Load and not the LA-Load method changes showed a significant relationship with performance changes. This is a novel finding that seems useful for taper planning of swimmers. Furthermore, training load changes are perceived correctly by the coach during taper but not during the normal training period.

Previous studies with swimmers of similar age have reported improvement from 1.6 to 5.2% after tapering of 11 or 28 days respectively (D’Aquisto et al., 1992; Papoti et al., 2007). Other studies show no improvement in performance after a taper of 7 or 14 days duration (Hopper et al., 1998). It should be noted that the data used to compare the performance changes in the present study were collected in official competitions where the season’s best performance as well as the performance before the start of the taper period were compared to NC performance. In contrast, previous studies compared the performance changes based on tests within the training session, while these tests were completed before the start and after the taper period (D’Aquisto et al., 1992; Hopper et al., 1998; Papoti et al., 2007). Swimmers may underperform during a testing session compared to a competition and this is critical for performance comparisons. It is also expected that percentage improvement of performance after a taper will be greater when the baseline for comparison is the pre-taper rather than the season’s best performance. This is because the pre-taper period is normally characterized by intensified training and a drop of performance as a consequence is expected. This is confirmed in the present study, since the improvement of performance in the NC compared to the season best was lower compared to performance change pre vs. post-taper (0.1 vs. 0.9%).

Several factors may affect the change of competitive performance following a taper. The training load is the most important factor and this is the product of training volume, intensity and frequency. It has been shown that the training load should be reduced by more than 50% (Mujika and Padilla, 2003) and training volume by 41 to 60% for an effective taper (Bosquet et al., 2007). In the present study the taper planned by the coaches failed to meet these criteria since the training load and volume were decreased only by 30% and 35% respectively from week 4 to the last week of taper. It should be noted that five of the six swimmers who improved their performance showed a greater difference in RPE-load between week 4 and week 1 (Figure 3). Although not significant, this 1.3% improvement in the performance of six swimmers may be important for getting a better place in the race. Previous studies have shown that performance changes of about 0.5% may affect the placing of young swimmers in a race (Stewart and Hopkins, 2000).

It is likely that the swimmers were not overloaded enough the weeks preceding the taper (weeks 3 and 4). In fact, the calculated training load was somewhat reduced during week 3 before a regional competition. It has been reported that performance may improve more after the taper with prior increased training overloading, than without prior overloading (Thomas et al., 2008). Furthermore, after reduction of the training load for a conference competition, the performance achieved in a following taper is reduced (Trinity et al., 2008). A likely suboptimal overload before the taper or a small percentage decrease of training load and distance from week 4 to week 1 before the NC may partly explain the failure to improve performance in this group of swimmers.

Previous studies have found improvement in mean tethered force measured during a 30 s test (3.8%) and peak force (11%) during 20 strokes of maximum intensity (Hooper et al., 1998; Papoti et al., 2007). In both studies the swimmers were tested after an intensified training period of four weeks and the tethered force was compared to that measured a day before or two days after the competition. In the present study no intensified training was followed before the taper and the swimmers were tested six days before the NC. Furthermore, different test duration (30 vs. 15 s) or different aspects of force (peak vs. mean force) were measured in previous studies (Hooper et al., 1998; Papoti et al., 2007). It is likely that force alterations occurred during the last five days of the taper and were not detected by the present experimental design. Probably, it is the ability for power production that may accompany any performance changes and this is more important than force changes.

All swimmers completed a 400 m submaximal test during the three testing periods. D’ Acquisto et al. (1992) found no changes in blood lactate and HR following a 2 min submaximal swimming at a velocity of 0.9 m·s^−1^ before and after a taper period. Furthermore, blood pH, bicarbonate, blood lactate and HR following a 200 m swim at 90% intensity were unchanged during a fifteen day taper period (Costill et al., 1985). It should be noted, however, that a tendency for decreased lactate values was observed in the last study (Costill et al., 1985) as it was the case in the present study. However, previous studies have seen increased submaximal lactate values during the last week of the taper (Van Handel et al., 1988). It is likely that elite swimmers show different rate of changes compared to young swimmers. Stroke rate changes at the V4 intensity have been shown to relate with performance (Anderson et al., 2008). However, no relationship of this parameter was observed in the present study. Probably the short observation period does not allow substantial changes in these variables.

It seems that calculation of the training load with the session-RPE method takes into account the “global” feeling of fatigue of the swimmers. In support to the value of this method is a high correlation between the difference in RPE-Load of week 4 and week 1 and percentage changes in performance time. The relationship of improved psychometric parameters during a taper with performance of swimmers has been confirmed using a questionnaire to examine the profile of mood state (Hooper et al., 1999). Furthermore, the session-RPE method has been shown to correlate well with other methods that have been previously used for the estimation of training load, such as the HR-based method (Impellizzeri et al., 2004; Wallace et al., 2009). However, there is no published comparison between the LA-Load and session-RPE methods used in the present study. Nevertheless, the methods used in the present study showed a good relationship between them (r=0.80), being within the range reported for comparison between different load estimation methods (r=0.65 to r=0.85) (Impellizzeri et al., 2004; Wallace et al., 2009). This strong relationship between methods indicates that they are both measuring the same parameter and are appropriate for use in swimming training. However, the easier to calculate RPE-Load method showed a relationship with competitive performance changes, but not the LA-Load method. This may give a practical advantage of the RPE-Load over the LA-Load method. Metabolic changes in the last 30 days of the season may have lead to misjudgment of the lactate-based training load. However, this seems unlikely since blood lactate response at the standard intensity corresponding to V4 was unchanged during the same period. Furthermore, it is unlikely that a linear model, such as this used in the present study, could explain performance changes of swimmers. Probably a mixed non-linear model could offer a better prediction of performance in swimming (Mujika et al., 1996; Thomas et al., 2009).

The coach perceived a higher training load than the swimmers. This probably has an impact on the training design. The difference in perception was greater during the normal training period (i.e. week 4) and decreased during the taper. Probably the coach communicates more frequently with the swimmers during this period taking into account the personal perception of fatigue during training (Maglischo, 2003). However, misjudgment of the training load during week 4 may have driven the coach to design a longer taper or to reduce the training load during weeks 1 and 2. On the other hand, the coach felt that he had adequately intensified the training, while this was not the case according to the swimmers’ perception. In agreement with the present findings, previous studies have shown that the perception of effort of the coach is higher than that of the swimmer (Stewart and Hopkins, 1997) and this difference is more evident during sessions of high intensity (Wallace et al., 2009). The lower perception of swimmers may be attributed to the fact that they perform interval training repetitions at an intensity lower than the prescribed, although they comply with distance and interval duration (Stewart and Hopkins, 1997). Whatever the case, these limited data reflect the ability of one coach and should be viewed as a case study.

In summary, the use of performance-related tests do not detect changes of training load and performance occurring during a taper. The use of session-RPE training load may be a useful and simple tool not only for the evaluation of the training load but also for the improvement of the taper planning. Swimmers may easily express their perception of effort after training sessions and this information should be effectively used by the coach, especially during the taper period, in order to apply changes in the training load on the following days. Establishment of the greatest possible difference in the training load of week 4 vs. the last week before the competition may help in improving performance in some swimmers. Coaches should record the training load of individual swimmers during the season and especially the weeks before and during the taper in order to decide on the appropriate load changes for optimal performance.

## Figures and Tables

**Table 1:** Changes in mean tethered force, fatigue index during the tethered force test, hand grip strength of both arms, body fat and lean body mass content during the three testing periods (Test 1, Test 2, Test 3)

	Test 1	Test 2	Test 3
TF (N)	112±38	114±41	115±41
FI (%)	17±8	16±8	20±10
HG-R (kg)	35±11	34±11	34±12
HG-L (kg)	33±9	32±8	33±9
BF (%)	20.1±6.3	19.1±5.9	19.5±5.9
LBM (kg)	50.3±9.9	50.8±9.8	50.2±9.1

TF: mean tethered force 15 s, FI: fatigue index during the TF test, HG-R and HG-L: hand grip strength of right and left arm, BF: body fat, LBM: lean body mass

**Table 2:** Data collected during the 400 m submaximal intensity test performed during the three testing periods (Test 1, Test 2, Test 3)

		Distance during the 400 m submaximal test							

		50	100	150	200	250	300	350	400
Test 1	SR (cycles·min^−1^)	31±6	30±6	31±5	31±6	31±5	32±5^*^	33±4^*^	34±5^*^
	SL (m·cycle^−1^)	2.49±0.41	2.41±0.38	2.34±0.36	2.36±0.40	2.34±0.34	2.30±0.34^*^	2.24±0.3^*^	2.23±0.3^*^
	HR (b·min^−1^)	157±13	167±8	171±8	176±9	178±9	180±10	185±4	186±5^*^
	speed (m·s^−1^)	1.23±0.12	1.16±0.16^*#^	1.18±0.11^*#^	1.19±0.10^*#^	1.20±0.12^*#^	1.20±0.12^*^	1.21±0.11	1.23±0.13

Test 2	SR (cycles·min^−1^)	31±6	30±5	31±5	31±5	31±5	32±5^*^	33±5^*^	33±5^*^
	SL (m·cycle^−1^)	2.54±0.40	2.36±0.32	2.33±0.30	2.29±0.29	2.30±0.30	2.27±0.30^*^	2.27±0.29^*^	2.25±0.31^*^
	HR (b·min^−1^)	148±16	165±9	166±9	171±9	176±5	179±8	184±5	186±4^*^
	speed (m·s^−1^)	1.26±0.12	1.17±0.13^*#^	1.17±0.13^*#^	1.17±0.12^*#^	1.19±0.12^*#^	1.20±0.12^*^	1.21±0.11	1.23±0.12

Test 3	SR (cycles·min^−1^)	31±6	30±6	30±6	31±6	32±5	32±6^*^	32±5^*^	33±5^*^
	SL (m·cycle^−1^)	2.48±0.43	2.41±0.39	2.41±0.36	2.36±0.34	2.30±0.38	2.28±0.39^*^	2.31±0.42^*^	2.21±0.30^*^
	HR (b·min^−1^)	158±8	166±8	169±8	172±8	178±7	181±7	183±4	186±5^*^
	speed (m·s^−1^)	1.23±0.11	1.17±0.12^*#^	1.18±0.13^*#^	1.19±0.13^*#^	1.19±0.13^*#^	1.21±0.12^*^	1.22±0.11	1.22±0.11

SR: stroke rate, SL: stroke length, HR: heart rate, Test 1, Test 2, Test 3: test performed 34, 20, 6 days before the national competition respectively.

*: p<0.05 compared to the first 50 m for the speed, SL and HR, compared to first, second and third 50 m for the SR.

#: p<0.05 compared with the last 50 m.
